# Effect of single‐session transcranial direct current stimulation on cognition in Parkinson's disease

**DOI:** 10.1111/cns.13210

**Published:** 2019-08-19

**Authors:** Chi‐ieong Lau, Mu‐N Liu, Kae‐Chwen Chang, Anna Chang, Chyi‐Huey Bai, Ching‐Shiang Tseng, Vincent Walsh, Han‐Cheng Wang

**Affiliations:** ^1^ Dementia Center, Department of Neurology Shin Kong Wu Ho‐Su Memorial Hospital Taipei Taiwan; ^2^ Applied Cognitive Neuroscience Group, Institute of Cognitive Neuroscience University College London London UK; ^3^ College of Medicine Fu‐Jen Catholic University Taipei Taiwan; ^4^ Institute of Brain Science National Yang‐Ming University Taipei Taiwan; ^5^ Department of Psychiatry Taipei Veterans General Hospital Taipei Taiwan; ^6^ Department of Neurology, Memory and Aging Centre University of California, San Francisco San Francisco CA USA; ^7^ Department of Rehabilitation Shin Kong Wu Ho-Su Memorial Hospital Taipei Taiwan; ^8^ Endeavor Rehabilitation Clinic Taipei Taiwan; ^9^ Department of Neurology Shin Kong Wu Ho-Su Memorial Hospital Taipei Taiwan; ^10^ Department of Public Health College of Medicine, Taipei Medical University Taipei Taiwan; ^11^ College of Medicine National Taiwan University Taipei Taiwan; ^12^ College of Medicine Taipei Medical University Taipei Taiwan

**Keywords:** inhibitory control, memory, Parkinson's disease, tDCS

## Abstract

**Aims:**

Nonmotor symptoms (NMS) such as cognitive impairment and impulse‐control disorders in Parkinson's disease (PD) remain a therapeutic challenge. Transcranial direct current stimulation (tDCS) has emerged as a promising alternative, although its immediate effects on NMS have been less well defined. In this randomized, sham‐controlled, crossover study, we aimed to explore the single‐session tDCS effects on cognitive performance in PD.

**Methods:**

Ten nondemented patients with PD completed two sessions in counterbalanced order, receiving 20 minutes of either 2 mA anodal or sham tDCS over the left dorsolateral prefrontal cortex (DLPFC). During stimulation, they performed the visual working memory and go/no‐go tasks. Performance of the tasks was compared between the two conditions.

**Results:**

Single‐session anodal tDCS over the left DLPFC did not significantly improve cognitive tasks in PD compared with sham (*P* > .05).

**Conclusion:**

Single‐session tDCS is ineffective in improving visual working memory and inhibitory control in PD. Further research may worth exploring alternative tDCS parameters, ideally with repeated sessions and concomitant training.

## 1 INTRODUCTION

Parkinson's disease (PD) is a multisystem neurodegenerative disorder that is increasingly recognized in our aging population. Current therapy mainly involves dopamine replacement which increases the hampered dopamine level in the corticobasal ganglia‐thalamocortical loop caused by dopaminergic neuronal loss at the substantia nigra.^1^ Albeit being effective for the cardinal motor symptoms including tremor, bradykinesia, and rigidity, these treatments are often stumped by nonmotor symptoms contributed by nondopaminergic mechanisms.^2^

Transcranial direct current stimulation is a promising technique that offers noninvasive neuromodulation by delivering a low intensity of electrical current via the scalp.^3^ A growing number of studies, albeit with mixed results, have demonstrated the efficacy of anodal tDCS in enhancing cognitive performance, both acutely and in long‐term, when applied over the DLPFC in healthy subjects and patients with neuropsychiatric disorders.^4^ Limited tDCS studies in PD focused on the prolonged effects of multiple‐session, often combined with either cognitive or physical training, on cognition or PIGD.^5^, ^6^ So far only one study explored the immediate effect of single‐session tDCS on working memory.^7^ The goal of this study was to investigate whether a single session of anodal tDCS over the left DLPFC would enhance cognitive performance in patients with PD.

## 2 MATERIALS AND METHODS

### 2.1 Subjects

**Figure 1:**
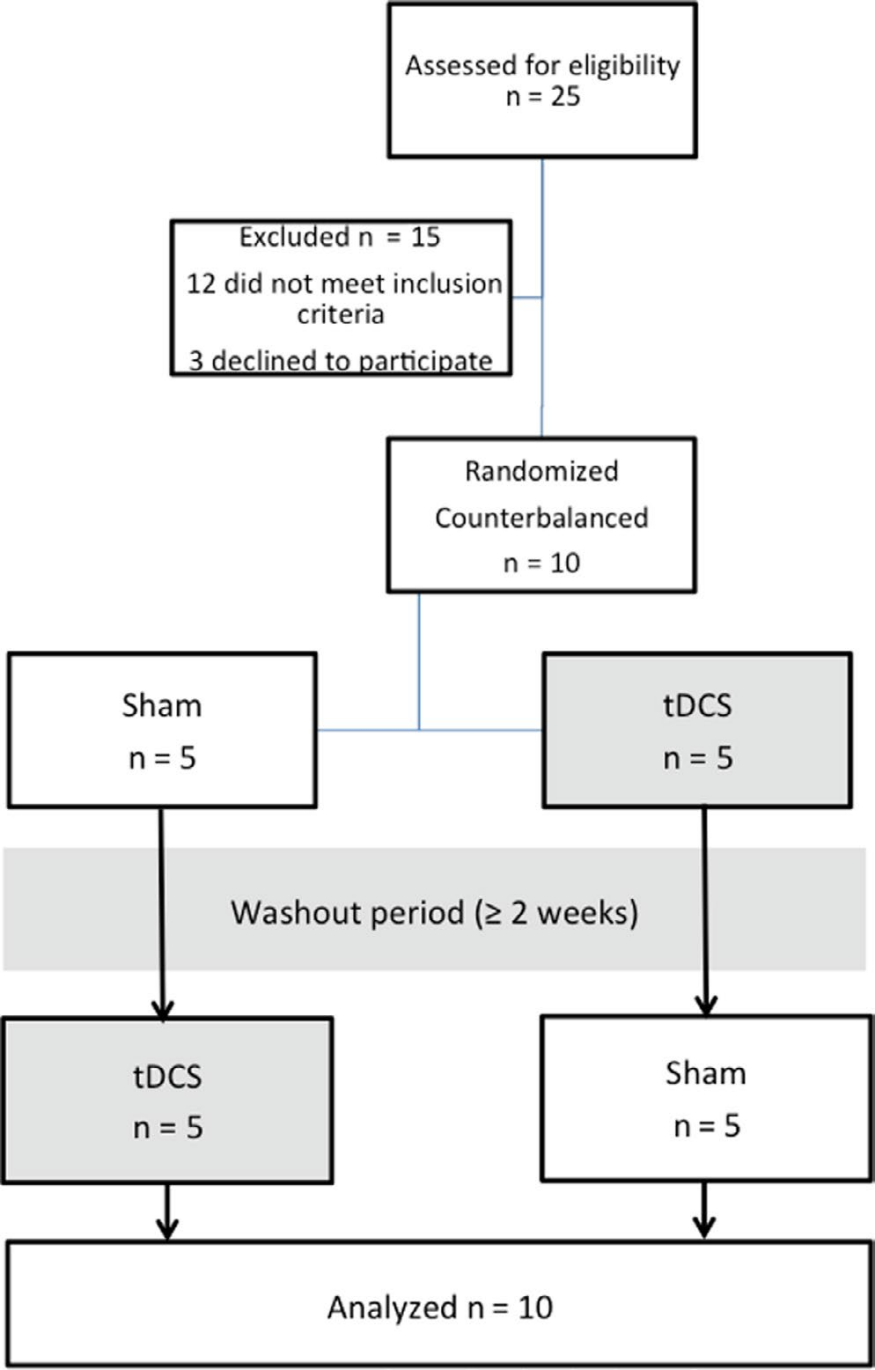
Flow diagram of the trial

A total of 10 idiopathic PD patients (5 men and 5 women), aged 56‐78 (mean 62.7 ± 6.6 years), who fulfilled the UK Parkinson's Disease Brain Bank criteria,^8^ were recruited for the study (Figure 1). All patients were requested to maintain stable dosages of levodopa and/or dopamine agonists and record medication diaries throughout the study.

Excluding criteria were (a) any known neurological or psychiatric disorders other than PD; (b) a history of head injury; (c) a history of seizure contradicting the use of tDCS; (d) patients who could not tolerate being withdrawn from antiparkinsonian medications for 12 hours; (e) patients with concomitant dementia with Mini‐Mental State Examination (MMSE) score less than 26 of 30. The study was approved by the Ethics Committee of the Shin‐Kong Wu Ho‐Su Memorial Hospital, and all subjects gave their informed consent.

### 2.2 Study design and experimental protocol

**Figure 2:**
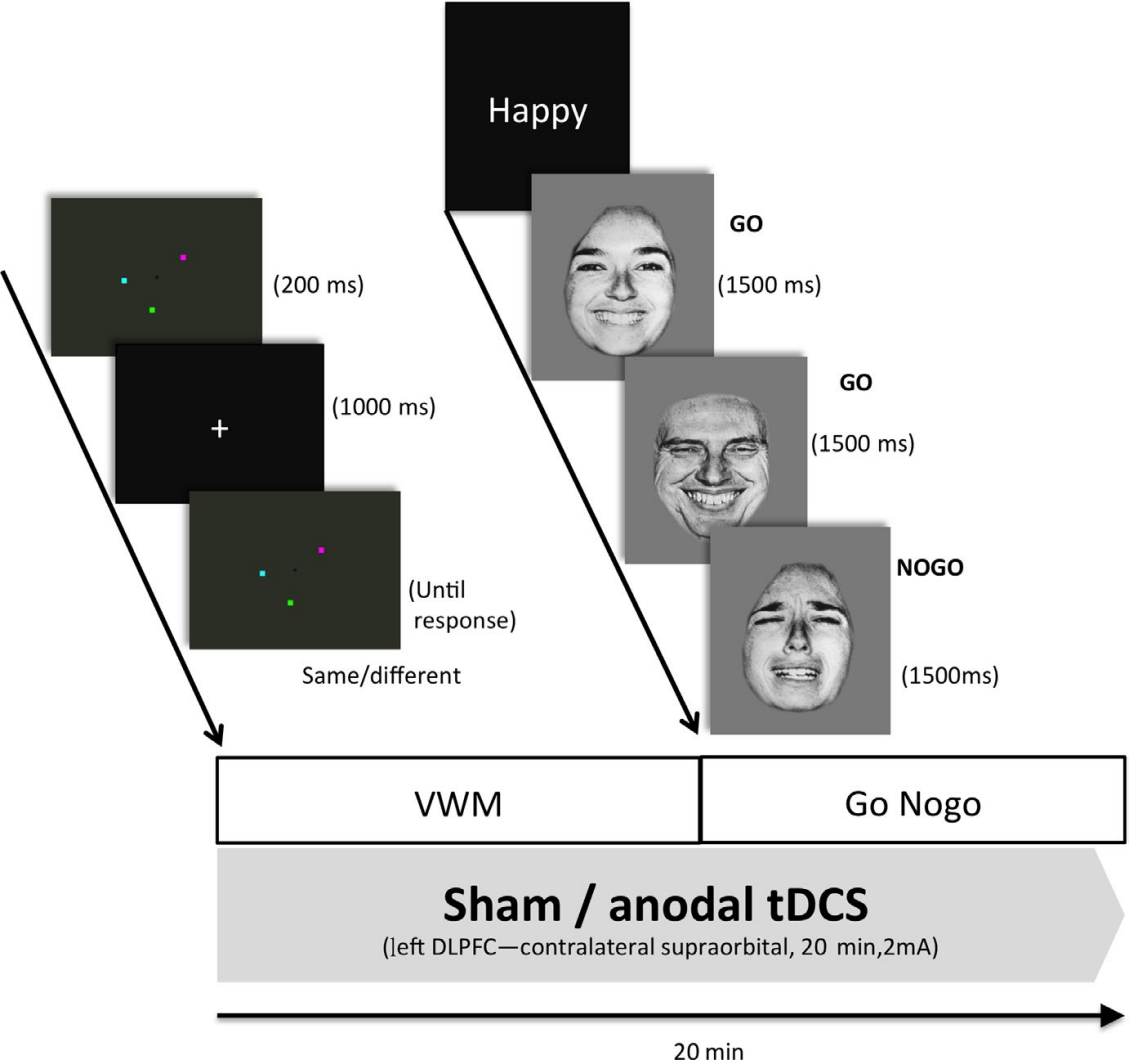
The protocol timeline. DLPFC, dorsolateral prefrontal cortex; VWM, visual working memory

Ten PD subjects attended 2 sessions during which either anodal tDCS or a sham intervention was applied to the left prefrontal cortex (Figure 1). The sessions in each subject were at least 2 weeks apart for a sufficient washout period. The stimulation order (tDCS/Sham) was counterbalanced across both sessions and blinded to the subject and clinical rater‐minimize potential biases. All subjects were requested to hold their antiparkinsonian medications for approximately 12 hours prior to the study. The experiments were conducted in the morning in order to minimize fluctuations in circadian rhythm. During tDCS/sham intervention, we tested two cognitive domains including (a) a visual working memory task and (b) a go/no‐go test to assess attention and response inhibition (Figure 2).

### 2.3 tDCS

A battery‐operated constant‐current DC‐Stimulator Plus (NeuroConn. Ilmenau, Germany) delivered a direct current of 2 mA via a saline‐soaked pair of sponge electrodes measuring 5 × 7 cm (35 cm^2^) to maximize the stimulation at the left DLPFC, the anode electrode was placed over F3 according to the 10—20 international system, whereas the cathode electrode at the contralateral (right) supraorbital area. Previous studies in healthy controls demonstrated improvement in cognitive performance with online anodal tDCS at the left DLPFC.^9^ For anodal tDCS, the current was applied for 20 minutes which initially ramped up over 10 seconds until reaching 2 mA. For sham tDCS, the electrode positions and stimulation parameters were the same as that used for anodal stimulation except that the current was delivered only for the initial 30 seconds. This produced the same temporary tingling sensation under the electrodes indistinguishable from the anodal tDCS, but without inducing effects on the brain.

### 2.4 Clinical assessments

#### 2.4.1 Visual working memory (VWM)—a change detection task

All subjects were seated at a distance of 80 cm in front of a 15‐inch computer screen in a quiet room. Each subject was given 10 trials of practice prior to the real task. A central fixation cross appeared at the beginning of each trial for 200 ms. This was immediately followed by a memory array of 3 different colored squares, randomly assigned in a given trial with no repetitive color in the same array. This memory array was displayed for 100 ms during which participants were asked to remember the colors. Following a 900 ms retention interval, another set of colored squares was presented until a response was given from the participant. They were instructed to identify whether the memory arrays were identical from the previous ones by pressing either the left (change) or right (no‐change) computer mouse button as fast as possible. Each patient was allowed to have 10 practice trials before recording their responses, that is accuracy and reaction time, in 4 separate blocks of 30 trials to determine the performance of change detection, we estimated d‐prime (d′) via average hit rate and false‐alarm rate. E‐prime 2.0 (Psychology Software Tools Inc) was used for both stimulus presentation and recording responses.

#### 2.4.2 Go/no‐go test

An emotional go/no‐go paradigm was used to evaluate response inhibition (impulsivity) of the participants. The task required subjects to press a button as fast as they could but to ensure accuracy when a displayed facial expression matched the preceding cue (Go cues) and to withhold pressing when the expression did not match the cue (Nogo cues). The set of stimuli included five facial expressions (happy, sad, fearful, angry, and neutral) of 10 adults (5 males and 5 females).^10^

Each trial began with a central fixation cross that appeared for 500 ms, followed by a randomly assigned cue (one of the five expressions, eg “Happy”) presented for 1000 ms, as the Go stimulus such that the remaining expressions (sad, fearful, angry, and neutral in this example) would be the Nogo stimuli. The Go stimuli comprised of 70% of each block to create a tendency for the subjects to respond. The experiment consisted of 240 trials split into 10 randomized blocks with each block covered 24 randomized trials of “Go—Nogo” pairs. Stimulus duration was 1500 ms. The first three blocks were used as practice trials.

#### 2.4.3 UPDRS‐III

At baseline, the motor part of the UPDRS was used to assess the motor severity by an independent neurologist.^8^ The motor subscores were calculated from the UPDRS‐III:

- Tremor: sum of items 20 (tremor at rest) and 21(action or postural tremor)
- Rigidity: item 22 (neck, and upper and lower body rigidity).
- Bradykinesia: sum of items 23 (finger tapping), 24 (hand open and closed), 25 (hand pronation/supination), and 26 (leg agility).
- PIGD: sum of items 27 (arising from chair), 28 (posture), 29 (gait), and 30 (postural stability).
- Speech and facial expression: sum of items 18 (speech) and 19 (facial expression).

### 2.5 Data analysis

To compare the two conditions (atDCS vs sham) on various outcome measures, the Wilcoxon signed‐rank test was used for assessment. Statistical analyses were performed on IBM^®^ SPSS^®^ (version 21), and the level of significance was set at *P* < .05.

## 3 RESULTS

Table 1 summarizes the demographic and clinical features of our patients. All patients were moderately to severely affected by PD with mean Hoehn & Yahr scale of 2.15 ± 0.3. All completed the study without any adverse effects with intervention (anodal tDCS or sham). Most patients (80%) experienced the brief initial tingling sensation after both active and sham tDCS, which was comparable with the percentage reported by previous studies,^11^ but all tolerated the intervention well without pain or major discomfort. Importantly, none of the patients were able to distinguish between tDCS and sham stimulation.

**Table 1:** Clinical and demographic features

	Frequency/mean ± SD	Ranges
Gender (male/female)	5/5	
Age (y)	62.7 ± 6.6	56‐78
Years of education	12.5 ± 3.8	6‐16
Disease duration (y)	7.8 ± 3.6	5‐15
HY scale	2.15 ± 0.3	2‐3
UPDRS－III	28.3 ± 15.0	9‐55
Tremor	8.3 ± 6.77	0‐19
Rigidity	6.6 ± 2.84	2‐12
Bradykinesia	6.3 ± 5.44	2‐20
PIGD	4.1 ± 2.23	2‐8
MMSE	26.2 ± 0.4	26‐27

Abbreviations: HY, Hoehn & Yahr; MMSE, Mini‐Mental State Examination; PIGD: postural instability and gait difficulty; SD, standard deviation; UPDRS, Unified Parkinson Disease Rating Scale.

### 3.1 Cognitive performance

A Wilcoxon signed‐ranks test indicates that the ability of detecting change in the VWM task, estimated using d‐prime, was not significantly different between anodal tDCS and sham stimulation, *P* = .80, suggesting that there was no immediate effect on the performance of VWM task with tDCS (Table 2).

**Table 2:** Results of cognitive and motor performance with anodal tDCS and sham stimulation

		Anodal tDCS		Sham		*P*‐value
		Median	Mean ± SD	Median	Mean ± SD	
VWM (d′)		1.86	1.92 ± 0.28	1.96	1.99 ± 0.4	.80
Go/no‐go test	RT, ms	548.02	554.60 ± 60.83	561.99	553.27 ± 42.27	.87
Go trials	Correct hit rate, %	88.44	81.81 ± 15.67	88.44	81.59 ± 15.36	.78
No‐go trials	False‐alarm rate, %	9.38%	12.13 ± 5.75	13.13%	10.75 ± 8.11	.51

Note

*P* values were obtained by nonparametric Wilcoxon signed‐rank test.

Abbreviations: D′: d‐prime; RT: reaction time; SD: standard deviation; VWM: visual working memory.

For the go/no‐go test, mean reaction time (RT) showed no significant difference between the two conditions, *P* = .87. Mean accuracy was around 82% but again no significant difference was found between tDCS and sham stimulation, *P* = .78 (Table 2).

## 4 DISCUSSION

This study compared the cognitive performance of PD after a single session of anodal tDCS versus sham over the left DLPFC. We hypothesized that tDCS intervention at the prefrontal cortices would enhance cognition in PD. In contrast to our speculations and results of previous studies,^7^, ^12^ we found no significant effect of a single‐session tDCS compared to sham on visual working memory and inhibitory control, as indexed by the VWM and go/no‐go tasks.

### 4.1 Effects of a single‐session tDCS on visual working memory in PD

In healthy subjects, Fregni et al^9^ were among the first to show that a single session of anodal tDCS over the left DLPFC could improve verbal working memory. Since then, a growing number of studies explored this notion but revealed inconsistent results, with some meta‐analyses showing null effects of DLPFC tDCS on working memory^13^ while others disclosed modest effects on reaction time but not accuracy of the tasks.^4^, ^14^

Our results add to the literature of studying the effect of a single‐session anodal tDCS over the prefrontal cortex on working memory, specifically, in an elderly cohort with nondemented PD. We were unable to replicate the findings of Boggio et al^7^ who showed significant improvement of working memory with left DLPFC tDCS, raising questions about the efficacy of single‐session excitatory tDCS on enhancing working memory in PD patients.

Nevertheless, there are some methodological issues that may worth mentioning. First, Boggio et al^7^ utilized a crossover design with memory assessment as the only behavioral task during (online) tDCS or sham intervention. Our study was also crossover designed but we simultaneously assessed two cognitive tasks during (online) intervention. Second, although the memory tasks in both studies were performed online, the previous study evaluated the performance in the last 5 minutes, whereas our memory task was assessed at the beginning of the 20‐minute intervention. Notably, Ohn and coworkers found a time‐dependent effect of anodal prefrontal tDCS on N‐back task whereby effect developed gradually and became reliable only at the end of the 30‐minute stimulation period.^15^ By contrast, another study demonstrated enhancement of working memory as early as 10 minutes of anodal tDCS.^9^

Third, for working memory assessment, the previous study utilized a three‐back task, whereas we used a visual change detection task. Recent studies revealed that visual working memory deficits in PD may be related to diminished storage capacity and impairment in filtering irrelevant information.^16^, ^17^ Tseng and colleagues showed improved visual change detection in low‐performing healthy subjects by applying anodal tDCS over the posterior parietal cortex.^18^ It may be argued that the neural correlates of visual change detection depend on the parietal cortex,^19^ yet other neuroimaging studies also emphasized the role of the frontal cortex in this task.^20^, ^21^ Likewise, whether active stimulation over the posterior parietal cortex or the left DLPFC would offer differential effects to various domains of working memory is still obscure. However, similar attempts at the left DLPFC have been made for the verbal domain of working memory and were able to induce improvement.^22^ Interestingly, neuroimaging studies reported that both n‐back test, either verbal or non‐verbal, and visual change detection task are associated with similar fronto‐parietal involvement,^23^ suggesting that similar neural correlates may underlie the mechanistic networks of both memory tasks.

Fourth, our study cohort showed comparable mean age (62.7 ± 6.6 vs 59.2 ± 9.9 years) and Hoehn and Yahr scale (2.15 ± 0.3 vs 2.3 ± 0.9) with that of Boggio.^7^ Although both cohorts were nondemented PD subjects with similar age and stage of disease, our subjects were generally higher in education (12.5 ± 3.8 vs 4.7 ± 4.4 years) and MMSE score (26.2 ± 0.4 vs 24.4 ± 3.1). Tseng and colleagues showed that only low performers benefited from tDCS in the visual change detection task while high performers did not, reflecting the possibility of a ceiling effect in this particular task.^18^ However, a subanalysis (not shown here) of our subjects showed that neither low nor high performers had significantly different results between tDCS and sham interventions.

Taken together, our results did not support the findings of Boggio et al^7^ and suggested that a single session of anodal tDCS may be insufficient to exert robust effect on working memory in PD. Current understanding of tDCS mechanisms proposes that acute modulation of neuronal resting membrane potential of the motor cortex via a polarity‐specific manner may underpin the acute effect of tDCS, whereby anodal and cathodal stimulation promotes neuronal depolarization or hyperpolarization, respectively.^24^ On the other hand, the enduring synaptic after‐effects of tDCS may share similarities with NMDA‐dependent long‐term potentiation and long‐term depression‐like cortical plasticity.^25^ In light of these proposed mechanisms, tDCS may be seen as an enhancer of synaptic plasticity. Repeated tDCS sessions, ideally combining with cognitive training as a learning process, may be necessary to achieve robust cognitive improvement.

### 4.2 No effect of a single‐session tDCS on impulsivity in PD

In addition to memory deficits, cognitive impairment in PD typically involves attention and executive dysfunction.^26^ One component of executive function is the ability to inhibit prepotent responses.^27^ Deficit in response inhibition, causing motor and behavioral impulsivity, often has negative impact on quality of life in PD. Emerging evidence suggests that prefrontal cholinergic circuits are critical for response inhibition in PD.^28^ In addition, patients may exhibit subclinical impairment in recognizing others' facial emotion, leading to biased emotional judgments.^29^ Considerable amount of evidence suggests that this facial emotional recognition deficit in PD may be explained by neural changes in a vast network of brain regions implicated in emotional processing and facial recognition—namely the ventrolateral prefrontal cortex, orbitofrontal cortex, striatum, amygdala, fusiform gyrus, and superior temporal sulcus.^30^, ^31^, ^32^

The go/no‐go task is frequently used to measure the ability to inhibit previously learned responses.^33^ One such task is the emotional go/no‐go task which has been widely used to test emotional processing in both healthy subjects and patients with affective disorders.^34^ This modified version of the task showing emotional faces may be considered as an affective set‐shifting task, simulating real‐life behavioral response toward recognizing emotions from faces.^33^ Thus, emotional go/no‐go task offers the advantage of simultaneously testing both behavioral inhibition and emotional processing, supporting its role in testing PD cohort with impulsivity and potential emotion recognition bias.

In healthy subjects, a decrease in left DLPFC activity induced by inhibitory rTMS resulted in impairment of the emotional go/no‐go task.^35^ Attempt has been made with anodal tDCS over the left DLPFC to increase the number of correct responses of the task in patients with major depression.^36^ On the other hand, anodal tDCS over the right DLPFC has also been demonstrated to enhance inhibitory control in the stop‐signal task,^37^ suggesting a plausible inhibitory link between the left and right DLPFC via transcallosal connection. Based on these preliminary findings, we hypothesized that excitatory tDCS over the left DLPFC may improve affective inhibition control in PD. Electric field induced by this montage may also reach deeper brain regions such as the orbitofrontal cortex, amygdala, and striatum which are involved in emotional processing.^38^

Our result, however, showed no significant difference in accuracy, false‐alarm rate, or reaction time of the go/no‐go task between active and sham tDCS in PD patients, suggesting that a single session of 2 mA anodal tDCS over the left DLPFC may not exert acute effect on affective inhibition control. Although being investigated in a few studies among healthy and other patient cohorts, the role of tDCS in modulating inhibitory control in PD patients has not yet been explored. It might be plausible that the null effect in our study was due to the differences in tDCS dose (2 mA vs 1.5 mA^39^), stimulating site (left vs right DLPFC^37^) or location of the returning electrode (contralateral vs. ipsilateral frontopolar region^36^). It might also reflect distinct fronto‐striatal neural mechanisms for impulsivity in PD compared with other study cohorts. Future studies, using a randomized controlled design, investigating the tDCS dose and montage‐dependent effects are needed to elucidate this assumption.

Intriguingly, our cohort of PD subjects seemed to perform quite well in facial emotional recognition, with a median of 88.44% of correct hit rate of the emotional go/no‐go task in both active and sham tDCS groups. This may be explained by the absence of concomitant dementia in our subjects. Indeed, recent studies indicate that facial emotional recognition deficit in PD may correlate positively with cognitive impairment.^29^

## 5 CONCLUSION

Taken together, our findings suggested that a single session of anodal tDCS over the left DLPFC is insufficient to improve working memory and inhibition control in patients with PD. However, with a small sample size, the results of this study should be interpreted with caution. Future studies with repeated tDCS sessions, coupling with cognitive and physical training, may be warranted to achieve sustainable facilitation of neuroplasticity.

## CONFLICT OF INTEREST

The authors declare no conflict of interest.

## ETHICAL APPROVAL

The study was approved by the Ethics Committee of the Shin‐Kong Wu Ho‐Su Memorial Hospital, and all subjects gave their informed consent.

## References

[1] Braak H , Ghebremedhin E , Rub U , Bratzke H , Del Tredici K . Stages in the development of Parkinson's disease‐related pathology. Cell Tissue Res. 2004;318:121‐134. 15338272 10.1007/s00441-004-0956-9

[2] Bohnen NI , Albin RL . The cholinergic system and Parkinson disease. Behav Brain Res. 2011;221:564‐573. 20060022 10.1016/j.bbr.2009.12.048 PMC2888997

[3] Shin YI , Foerster A , Nitsche MA . Transcranial direct current stimulation (tDCS) ‐ application in neuropsychology. Neuropsychologia. 2015;69:154‐175. 25656568 10.1016/j.neuropsychologia.2015.02.002

[4] Dedoncker J , Brunoni AR , Baeken C , Vanderhasselt MA . A systematic review and meta‐analysis of the effects of Transcranial Direct Current Stimulation (tDCS) over the dorsolateral prefrontal cortex in healthy and neuropsychiatric samples: influence of stimulation parameters. Brain Stimul. 2016;9:501‐517. 27160468 10.1016/j.brs.2016.04.006

[5] Dinkelbach L , Brambilla M , Manenti R , Brem AK . Non‐invasive brain stimulation in Parkinson's disease: exploiting crossroads of cognition and mood. Neurosci Biobehav Rev. 2017;75:407‐418. 28119070 10.1016/j.neubiorev.2017.01.021

[6] Broeder S , Nackaerts E , Heremans E , et al. Transcranial direct current stimulation in Parkinson's disease: Neurophysiological mechanisms and behavioral effects. Neurosci Biobehav Rev. 2015;57:105‐117. 26297812 10.1016/j.neubiorev.2015.08.010

[7] Boggio PS , Ferrucci R , Rigonatti SP , et al. Effects of transcranial direct current stimulation on working memory in patients with Parkinson's disease. J Neurol Sci. 2006;249:31‐38. 16843494 10.1016/j.jns.2006.05.062

[8] Goetz CG , Tilley BC , Shaftman SR , et al. Movement Disorder Society‐sponsored revision of the Unified Parkinson's Disease Rating Scale (MDS‐UPDRS): scale presentation and clinimetric testing results. Mov Disord. 2008;23:2129‐2170. 19025984 10.1002/mds.22340

[9] Fregni F , Boggio PS , Nitsche M , et al. Anodal transcranial direct current stimulation of prefrontal cortex enhances working memory. Exp Brain Res. 2005;166:23‐30. 15999258 10.1007/s00221-005-2334-6

[10] Zelano J . Poststroke epilepsy: update and future directions. Ther Adv Neurol Disord. 2016;9:424‐435. 27582897 10.1177/1756285616654423 PMC4994782

[11] Kessler SK , Turkeltaub PE , Benson JG , Hamilton RH . Differences in the experience of active and sham transcranial direct current stimulation. Brain Stimul. 2012;5:155‐162. 22037128 10.1016/j.brs.2011.02.007 PMC3270148

[12] Lattari E , Costa SS , Campos C , de Oliveira AJ , Machado S , Maranhao Neto GA . Can transcranial direct current stimulation on the dorsolateral prefrontal cortex improves balance and functional mobility in Parkinson's disease? Neurosci Lett. 2017;636:165‐169. 27838447 10.1016/j.neulet.2016.11.019

[13] Hill AT , Fitzgerald PB , Hoy KE . Effects of anodal transcranial direct current stimulation on working memory: a systematic review and meta‐analysis of findings from healthy and neuropsychiatric populations. Brain Stimul. 2016;9:197‐208. 26597929 10.1016/j.brs.2015.10.006

[14] Brunoni AR , Vanderhasselt MA . Working memory improvement with non‐invasive brain stimulation of the dorsolateral prefrontal cortex: a systematic review and meta‐analysis. Brain Cogn. 2014;86:1‐9. 24514153 10.1016/j.bandc.2014.01.008

[15] Ohn SH , Park CI , Yoo WK , et al. Time‐dependent effect of transcranial direct current stimulation on the enhancement of working memory. NeuroReport. 2008;19:43‐47. 18281890 10.1097/WNR.0b013e3282f2adfd

[16] Zhao G , Chen F , Zhang Q , Shen M , Gao Z . Feature‐based information filtering in visual working memory is impaired in Parkinson's disease. Neuropsychologia. 2018;111:317‐323. 29427571 10.1016/j.neuropsychologia.2018.02.007

[17] Lee EY , Cowan N , Vogel EK , Rolan T , Valle‐Inclán F , Hackley SA . Visual working memory deficits in patients with Parkinson's disease are due‐both reduced storage capacity and impaired ability‐filter out irrelevant information. Brain. 2010;133:2677‐2689. 20688815 10.1093/brain/awq197 PMC2929336

[18] Tseng P , Hsu TY , Chang CF , et al. Unleashing potential: transcranial direct current stimulation over the right posterior parietal cortex improves change detection in low‐performing individuals. J Neurosci. 2012;32:10554‐10561. 22855805 10.1523/JNEUROSCI.0362-12.2012 PMC6621415

[19] Beck DM , Muggleton N , Walsh V , Lavie N . Right parietal cortex plays a critical role in change blindness. Cereb Cortex. 2006;16:712‐717. 16120797 10.1093/cercor/bhj017

[20] Turatto M , Sandrini M , Miniussi C . The role of the right dorsolateral prefrontal cortex in visual change awareness. NeuroReport. 2004;15:2549‐2552. 15538193 10.1097/00001756-200411150-00024

[21] Huettel SA , Guzeldere G , McCarthy G . Dissociating the neural mechanisms of visual attention in change detection using functional MRI. J Cogn Neurosci. 2001;13:1006‐1018. 11595102 10.1162/089892901753165908

[22] Javadi AH , Walsh V . Transcranial direct current stimulation (tDCS) of the left dorsolateral prefrontal cortex modulates declarative memory. Brain Stimul. 2012;5:231‐241. 21840287 10.1016/j.brs.2011.06.007

[23] Pessoa L , Ungerleider LG . Neural correlates of change detection and change blindness in a working memory task. Cereb Cortex. 2004;14:511‐520. 15054067 10.1093/cercor/bhh013

[24] Nitsche MA , Fricke K , Henschke U , et al. Pharmacological modulation of cortical excitability shifts induced by transcranial direct current stimulation in humans. J Physiol. 2003;553:293‐301. 12949224 10.1113/jphysiol.2003.049916 PMC2343495

[25] Monte‐Silva K , Kuo MF , Hessenthaler S , et al. Induction of late LTP‐like plasticity in the human motor cortex by repeated non‐invasive brain stimulation. Brain Stimul. 2013;6:424‐432. 22695026 10.1016/j.brs.2012.04.011

[26] Kudlicka A , Clare L , Hindle JV . Executive functions in Parkinson's disease: systematic review and meta‐analysis. Mov Disord. 2011;26:2305‐2315. 21971697 10.1002/mds.23868

[27] Frank MJ , Samanta J , Moustafa AA , Sherman SJ . Hold your horses: impulsivity, deep brain stimulation, and medication in parkinsonism. Science. 2007;318:1309‐1312. 17962524 10.1126/science.1146157

[28] Ye Z , Altena E , Nombela C , et al. Selective serotonin reuptake inhibition modulates response inhibition in Parkinson's disease. Brain. 2014;137:1145‐1155. 24578545 10.1093/brain/awu032 PMC3959561

[29] Argaud S , Verin M , Sauleau P , Grandjean D . Facial emotion recognition in Parkinson's disease: a review and new hypotheses. Mov Disord. 2018;33:554‐567. 29473661 10.1002/mds.27305 PMC5900878

[30] Yoshimura N , Kawamura M , Masaoka Y , Homma I . The amygdala of patients with Parkinson's disease is silent in response‐fearful facial expressions. Neuroscience. 2005;131:523‐534. 15708493 10.1016/j.neuroscience.2004.09.054

[31] Wabnegger A , Ille R , Schwingenschuh P , et al. Facial emotion recognition in parkinson's disease: an fMRI investigation. PLoS ONE. 2015;10:e0136110. 26285212 10.1371/journal.pone.0136110 PMC4540566

[32] Le Jeune F , Peron J , Biseul I , et al. Subthalamic nucleus stimulation affects orbitofrontal cortex in facial emotion recognition: a PET study. Brain. 2008;131:1599‐1608. 18490359 10.1093/brain/awn084 PMC2408938

[33] Schulz KP , Fan J , Magidina O , Marks DJ , Hahn B , Halperin JM . Does the emotional go/no‐go task really measure behavioral inhibition? Convergence with measures on a non‐emotional analog. Arch Clin Neuropsychol. 2007;22:151‐160. 17207962 10.1016/j.acn.2006.12.001 PMC2562664

[34] Meule A . Reporting and interpreting task performance in go/no‐go affective shifting tasks. Front Psychol. 2017;8:701. 28536544 10.3389/fpsyg.2017.00701 PMC5422529

[35] Bermpohl F , Fregni F , Boggio PS , et al. Left prefrontal repetitive transcranial magnetic stimulation impairs performance in affective go/no‐go task. NeuroReport. 2005;16:615‐619. 15812319 10.1097/00001756-200504250-00020

[36] Boggio PS , Bermpohl F , Vergara AO , et al. Go‐no‐go task performance improvement after anodal transcranial DC stimulation of the left dorsolateral prefrontal cortex in major depression. J Affect Disord. 2007;101:91‐98. 17166593 10.1016/j.jad.2006.10.026

[37] Hsu TY , Tseng LY , Yu JX , et al. Modulating inhibitory control with direct current stimulation of the superior medial frontal cortex. NeuroImage. 2011;56:2249‐2257. 21459149 10.1016/j.neuroimage.2011.03.059

[38] Bai S , Dokos S , Ho KA , Loo C . A computational modelling study of transcranial direct current stimulation montages used in depression. NeuroImage. 2014;87:332‐344. 24246487 10.1016/j.neuroimage.2013.11.015

[39] Soltaninejad Z , Nejati V , Ekhtiari H . Effect of anodal and cathodal transcranial direct current stimulation on DLPFC on modulation of inhibitory control in ADHD. J Atten Disord. 2019;23:325‐332. 26689935 10.1177/1087054715618792

